# Effects of Heat-Killed *Lactococcus lactis* Strain Plasma on Skin Homeostasis-Related Genes and the Skin Microbiome among Healthy Adults: A Randomized Controlled Double-Blind Study

**DOI:** 10.3390/microorganisms9102029

**Published:** 2021-09-25

**Authors:** Toshio Fujii, Takashi Fujitomo, Ryohei Tsuji, Ryuichi Kubo, Yukiko Kato, Osamu Kanauchi

**Affiliations:** 1 Research Laboratories for Health Science & Food Technologies, Kirin Holdings Co., Ltd., Yokohama 2360004, Japan; Ryohei_Tsuji@kirin.co.jp (R.T.); Yukiko_Kato@kirin.co.jp (Y.K.); kanauchio@kirin.co.jp (O.K.); 2 DeNA Life Science Inc. R&D Group, Tokyo 1608582, Japan; t.fujitomo@gmail.com (T.F.); rkubo.4w@gmail.com (R.K.)

**Keywords:** *Lactococcus lactis* subsp. *lactis* strain LC-plasma, immunity, plasmacytoid dendritic cells, skin microbiome, tight junction, antimicrobial peptides, skin homeostasis

## Abstract

*Lactococcus lactis* subsp. *lactis* strain plasma (LC-plasma) is a bacterial strain that activates plasmacytoid dendritic cells and induces viral resistance genes via the TLR9/MyD88 pathway. We recently showed that oral administration of LC-plasma prevents skin infection by *Staphylococcus aureus*, possibly by activating skin immunity. In this study, we conducted a double-blind clinical trial to investigate the effect of oral administration of heat-killed LC-plasma on the skin microbiome, gene expression in the skin, and the skin condition of healthy volunteers. Seventy healthy volunteers were randomly assigned to receive either heat-killed LC-plasma or a placebo for eight weeks. Analysis of the skin microbiome by next-generation sequencing suggested that the alpha-diversity of the skin microbiome did not change during the test period in either group. However, the proportion of species that changed significantly during the test period was 10-fold smaller in the LC-plasma group than in the placebo group, suggesting that LC-plasma may maintain the skin microbiome. Quantitative PCR analysis indicated that tight-junction genes, such as *CLDN1* and *CLDN12*, and the antimicrobial peptide gene *BD3* were significantly up-regulated in the LC-plasma group but not in the placebo group. Our results suggest that administration of LC-plasma helps to maintain the skin microbiome and that it affects homeostasis-related genes.

## 1. Introduction

Lactic acid bacteria (LAB) are widely accepted as beneficial microorganisms for human health. Recent scientific studies have revealed that not only living cells but also dead cells of LAB, termed paraprobiotics, have multiple beneficial effects [1,2]. Paraprobiotics have a large advantage over probiotics in terms of commercial use. They come in a wide variety of formulations, have a relatively long shelf-life and stable quality control, and are easy to handle, transport, and store [3,4]. In addition, paraprobiotics have no risk of infection in the human body. Many types of supplements, food, and beverages containing paraprobiotics have been marketed in the past several years.

We have carried out a number of studies of the paraprobiotics strain *Lactococcus lactis* subsp. *lactis* strain plasma (LC-plasma). Initially, we found that heat-killed LC-plasma cells are directly engulfed by plasmacytoid dendritic cells (pDCs), which subsequently activate B-cells, T-cells, and natural killer (NK) cells [5]. Subsequently, we showed that the genomic DNA of LC-plasma activates pDCs via the TLR9-MyD88 pathway [6]. Our bioinformatics analysis suggested that LAB strains containing a high copy number of CpG motifs in the AT-rich (>40%) region of the genome show high pDC stimulatory activity and that LC-plasma is one of the strains that has the highest copy numbers of CpG motifs in this region [7]. We have also performed human clinical trials, which showed that LC-plasma increases the activity of pDCs in peripheral blood mononuclear cells (PBMCs) as well as systemic and local immunity, reduces the risk of infection, and alleviates the symptoms of the common cold or seasonal influenza [8,9,10]. Interestingly, our most recent non-clinical study in mice suggested that long-term administration of LC-plasma maintained skin thickness and a healthy coat of fur in the latter half of life [11].

Skin is the largest organ, covering approximately 1.5–2.0 m^2^, and it functions as a physical barrier that protects the body from environmental threats, such as pathogen infection, solar light, and dryness. The skin barrier is composed of several defense systems, including skin immunity, tight junctions (TJs), and antimicrobial peptides (AMPs). Recent evidence suggests that these defense systems are engaged in cross-talk with the commensal microbiota on the skin surface [12]. Skin commensal microorganisms increase the production of interleukin 1 (IL-1), which is involved in initiating and amplifying skin immune systems. These commensal bacteria also modulate the function of local T cells and promote both host defense and inflammatory diseases associated with high levels of IL-17A [13].

Antimicrobial peptides are small cationic peptides that are mainly produced by epithelial cells and have pleiotropic bactericidal effects. *Staphylococcus epidermidis*, one of the major skin commensal bacteria, has been reported to increase the expression of β-defensin genes [14]. Tight junctions provide a barrier to prevent the passage of both ions and molecules through the paracellular pathway. Claudin and occludin have been identified as major components of TJs. Disorders in TJs lead to skin diseases such as atopic dermatitis and acne, which are associated with the overgrowth of *Staphylococcus aureus* and *Propionibacterium acnes*, respectively. To date, however, knowledge about the effects of probiotics and paraprobiotics on skin immunity, TJ genes, AMP genes, and cutaneous microbiota remains limited.

To elucidate the oral administration effect of LC-plasma on skin defense systems, we previously conducted a nonclinical study in which LC-plasma was found to activate the pDCs of skin-draining lymph nodes and increase the production of IL-1α in dorsal skin. Interestingly, the expression level of TJ genes (*Cldn1* and *Tjp1*) and AMP genes (*Defb1, Defb2*, *Defb3*, *Defb4*, and *S100a8*) were significantly higher in the LC-plasma group than in the control group [15]. Furthermore, the skin extract of LC-plasma-treated mice showed antibacterial activity against *S. aureus* and *P. acnes* [15]. Moreover, when the mouse skin was challenged with a δ-toxin-producing strain of *S. aureus*, oral administration of LC-plasma suppressed the growth of *S. aureus* and alleviated the skin inflammation caused by the bacterial toxin [15]. Taken together, these findings strongly suggest that oral administration of LC-plasma affects the skin defense systems and protects against external environmental threats, such as bacterial infections.

The aim of this study was to evaluate the effect of LC-plasma on skin condition in humans via a double-blind clinical trial among healthy volunteers. The effects of LC-plasma on the skin microbiome, which provides an important barrier to defend the skin against microbial infection, were studied. We also studied the effects of oral administration of LC-plasma on the expression level of immunity genes, TJ genes, and AMP genes in the skin. Lastly, we evaluated the effect of LC-plasma on levels of hydration and pigmentation, which have been reported to be associated with the skin microbiome and probiotics administration [16,17].

## 2. Materials and Methods

### 2.1. Subjects

The volunteers, recruited in December 2016, were healthy Japanese adults aged 30–60 years, and they did not meet the exclusion criteria (Table 1). Informed consent was obtained from all candidates after they were provided the full details of the study, in accordance with the Declaration of Helsinki (revised version of 2013). Individuals with a serious disease (e.g., immune-mediated disease, hepatic disorder, renal disorder, cardiac disease, anemia, and anamnesis), a skin disorder (e.g., severe atopic symptoms), or an allergy; those who were pregnant; and those routinely taking supplements containing immune-simulating or immune-suppressing products were excluded. Individuals with a skin disease requiring the application of medicine containing steroids or antibiotics to their face, those with severe menopausal symptoms, and those who had continuously taken medicines for skin condition improvement within the past 3 months were also excluded from the trial. In total, 71 volunteers were selected on the basis of the results from a pre-blood test (WBC, RBC, Hb, Ht, MCV, MCH, MCHC, Plt, neutrophil, eosinophil, basophil, lymphocyte, total-cholesterol, TG, LDL-cholesterol, HDL-cholesterol, BUN, CRE, UA, AST, ALT, γ-GT, LD, T-Bil, CK BS, HbA1c), a pDC activity test (CD86, HLA-DR), a skin moisture content analysis, and a background questionnaire.

Eligible subjects received their treatment pack consisting of one capsule per day to be consumed each day for 8 weeks. During the test period, subjects were prohibited from changing their lifestyle, including diet and cosmetics, and from using medicines, herbs, and functional foods, unless absolutely necessary. Subjects were prohibited from receiving food or medicine containing LAB. Subjects were asked to avoid sunburn and overseas trips. All eligible subjects were requested to keep a web-diary every day to assess compliance with capsule ingestion and compliance with instructions and to record the use of any concomitant medication.

### 2.2. Study Design

The study was designed on the basis of our previous study, in which we observed the effect of LC-plasma on immune activity [8]. A randomized, controlled, double-blind trial was conducted in which eligible subjects were randomized and allocated to two groups: a placebo group (36 adults) and the LC-plasma group (35 adults) using a stratified randomization method using an in-house R script. Gender, age (>40 or <40 years), pDC activity (CD86), and skin moisture content were used as stratification factors. The values of CD86 were determined as was previously described [9]. Subjects in the placebo group consumed silica-coated hard capsules containing 200 mg of non-genetically-engineered cornstarch, and those in the LC-plasma group consumed identical-looking capsules containing 50 mg (1 × 10^11^ cells) of heat-killed and spray-dried LC-plasma and 150 mg of non-genetically-engineered cornstarch for 8 weeks from January to March 2017. Neither the investigators nor the participants could differentiate between the placebo capsules and the LC-plasma capsules. Allocation was pre-assigned on the basis of randomization numbers and was concealed from the subjects and researchers. Furthermore, the capsule codes were not revealed until all analyses were complete and the data set was secured. The study was conducted at Medical Corporation Wakei-kai Medics Hongo Clinic (Tokyo, Japan), and the protocol was approved by the clinical research ethics committee of DeNA Life Science Inc (Tokyo, Japan).

The minimum sample size was determined on the basis of CD86 as a marker of pDC activity, because pDC is thought to be a direct target of LC-plasma. Our previous study reported a standard deviation for CD86 of 20%, with a difference in pre- and post-administration mean values of 15%. Considering these parameters, we estimated that a sample size of 29 subjects in each arm would be required to achieve at least 80% power (β ≥ 0.8) with statistical significance (α ≤ 0.05) in a paired t-test. The study was registered through the University Hospital Medical Information Network as UMIN 000025566.

### 2.3. Study Outcomes

The primary efficacy outcome measure was the composition of the skin microbiome based on 16S rDNA analysis. Secondary outcome measures were transcripts from skin-immunity-related genes, AMP genes, and skin homeostasis-related genes; TEWL; skin moisture content; and skin color values.

### 2.4. Preparation of Total RNA from Hair-Root

Because epidermal keratinocytes lose the nucleus [18], it was possible to purify a little amount of total RNA from skin stratum corneum. In addition, for ethical reasons, it is difficult to obtain biopsy samples from healthy humans’ faces. Given these factors, we isolated total RNA samples from scalp-hair root, as previously described [19]. In brief, 5–10 hairs were plucked from the scalp of each subject by using forceps. The hairs were checked for the presence of sheaths. Hairs containing the bulb region were trimmed to approximately 1.0 cm in length and immediately dipped into 1.0 mL of RNAlater (Thermo Fishcer Science, Waltham, MA, USA) in a 1.5-mL microtube. Total RNA was extracted from hair follicles by using an RNAqueous^®^-4PCR Kit (Thermo Fishcer Science, MA, USA), and cDNA was prepared by using an iScript cDNA synthesis kit (BioRad, Hercules, CA, USA) in accordance with the manufacturer’s protocol.

### 2.5. Quantitative PCR Analysis

Quantitative PCR (qPCR) was performed with SYBR Premix Ex Taq (TaKaRa Bio, Kusatsu, Japan) and a LightCycler 480 (Roche, Basel, Switzerland). The relative expression of each gene was determined in comparison with a reference gene, *ACTB* (β-actin), by using a relative standard curve method. The primers used for the qPCR analysis are listed in Table S1.

### 2.6. Skin Moisture, TEWL, and Pigmentation Assessment

Before skin examination, each subject washed their face and then rested for 20 min in a waiting room kept under mild environmental conditions (room temperature, 21 ± 1 °C; relative humidity, 50 ± 10%) in order to maintain consistent environmental and measurement conditions as far as possible. Skin moisture in the stratum corneum was measured by using a Corneometer^®^ CM825 instrument (Courage + Khazaka Electronic GmbH, Cologne, Germany) at the left cheek bone area. The reported values were the average of three measurements. 

TEWL scores (g/h/m^2^) were obtained by using a Tewameter^®^ TM300 instrument (Courage + Khazaka Electric GmbH, Köln, Germany) at the left cheek bone area. Pigmentation of skin equivalents was assessed by comparing the change in L *, a *, and b * values of CIE 1976 color space measured by a CM-2600d instrument (Konika minorta, Tokyo, Japan). L * represents brightness, with the darkest black at L * = 0 and the brightest white at L * = 100. a * represents the green–red component, with green in the negative direction and red in the positive direction. b * represents the blue–yellow component, with blue in the negative direction and yellow in the positive direction. The change in pigmentation was determined by calculating the difference between the mean at 8 weeks (8 W) and the mean at 0 weeks (0 W) for each skin equivalent. 

### 2.7. Skin Microbiome Sample Collection, DNA Extraction, and Metagenomics Shotgun Sequencing

Subjects were asked to collect skin microbiome samples from their cheek in the morning before attending the clinic; samples were collected before breakfast by wiping their faces using a sterilized cotton swab (Copan diagnostics, Murrieta, CA, USA) following the manufacturer’s instructions. The swab was immediately placed into a 1.0 mL sterile tube and kept at 4 °C. Samples were processed within 24 hours of collection.

The 16S rRNA gene was amplified by using an Ion 16S Metagenomics kit (Thermo Fisher Scientific). DNA samples were extracted by using a QIAamp UCP Pathogen Mini Kit. One nanogram of extracted genomic DNA was tagmented by using Ion Xpress Barcode Adaptors. Indexed libraries were amplified by using an Ion PGM Hi-Q View OT2 kit. About 400-bp DNA sequences were determined by using an Ion PGM Hi-Q View Sequencing Kit. All processes of DNA extraction, PCR amplification, next-generation sequencing, and bioinformatics analysis except linear discriminant analysis effect size (LEfSe) were performed by World Fusion, Co. Ltd. (Tokyo, Japan).

### 2.8. Bioinformatics Analysis

The determined 16S rDNA sequences were subjected to BLAST homology searching against the NCBI 16S Microbial database by using Metagenome@KIN software (World Fusion Co., Ltd., Tokyo, Japan). Microbial diversity was assessed by using the Simpson index and the Shannon–Weiner index, which accounts for both the number of phylotypes (richness) and the proportion of the total accounted for by each phylotype (evenness). LEfSe analysis was performed by using Galaxy v. 1 [20].

### 2.9. Statistical Analysis

Comparisons of continuous variables before and after intake of placebo and LC-plasma were performed by using a paired *t*-test. Non-paired t-tests were not used in this study primarily because the composition of the skin microbiome and the baseline gene expression levels had large variations among the healthy volunteers. If a significant (*p <* 0.05) or marginally significant (*p <* 0.10) difference was observed in the placebo or the LC-plasma group, the post-hoc test of delta value (0–8 W) was performed using the non-paired *t*-test. Bonferroni’s method was used to allow for multiple comparisons, and a significant *p* value was set at *p <* 0.05 / 2 = 0.025; marginal significance was set at *p <* 0.10 / 2 = 0.05. The relative quantity of the skin microbiome affected by LC-plasma was evaluated using the chi-square test. The results are expressed as means and standard deviations.

## 3. Results

### 3.1. Subject Characteristics

**Figure 1:**
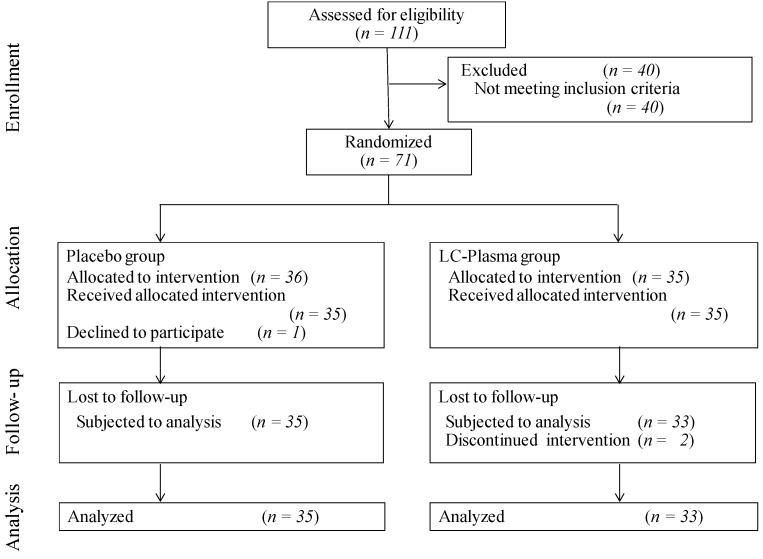
The consolidated standards of reporting trials (CONSORT) flow diagram.

The consolidated standards of reporting trials (CONSORT) flow diagram for the study is shown in Figure 1. Eligible subjects were randomized and allocated to two groups: the placebo group (36 adults) and the LC-plasma group (35 adults) by using a stratified randomization method as described in the Materials and Methods section. One subject in the placebo group declined to participate after allocation. During the intake period, two subjects from the LC-plasma group were lost to follow-up due to personal commitments on the sampling day. As a result, data from 35 volunteers in the placebo group and 33 in the LC-plasma group were included in the analysis. The baseline characteristics of the participants are summarized in Table 2. No adverse events attributed to the test supplement were observed during the test period.

### 3.2. Alpha Diversity of the Skin Microbiome

**Figure 2:**
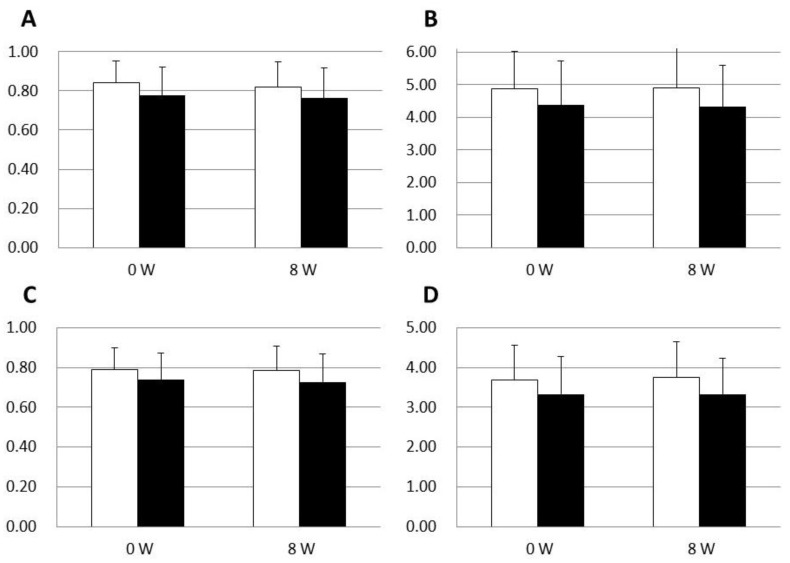
Comparison of Simpson and Shannon–Weiner indexes. Open rectangles indicate the placebo group, and filled rectangles indicate the LC-plasma group. (**A**) Simpson indexes of species. (**B**) Shannon–Weiner indexes of species. (**C**) Simpson indexes of genus. (**D**) Shannon–Weiner indexes of genus.

To evaluate the effect of LC-plasma on the skin microbiome of the cutaneous layer, we collected samples from the cheek using a swab. The quality-filtered and non-chimeric sequences for all samples were clustered into bins, termed operational taxonomic units (OTUs). The microbiome data were classified into bacterial taxa, from phyla to species. Indices of alpha diversity (Simpson and Shannon–Weiner indexes) were calculated for each species and genus. The results are shown in Figure 2. The mean Simpson index for species and genera was 0.84 and 0.79, respectively, in the placebo group, as compared with 0.79 and 0.74, respectively, in the LC-plasma group. These suggested that the microbiome of the volunteers was highly diverse in both groups. No significant change in the alpha diversity of species and genera was observed in either the placebo group or the LC-plasma group after the test period. Similarly, no significant difference in the Shannon–Weiner index was observed after the test period in either group.

### 3.3. Change in the Relative Quantity of Microbiota

**Figure 3:**
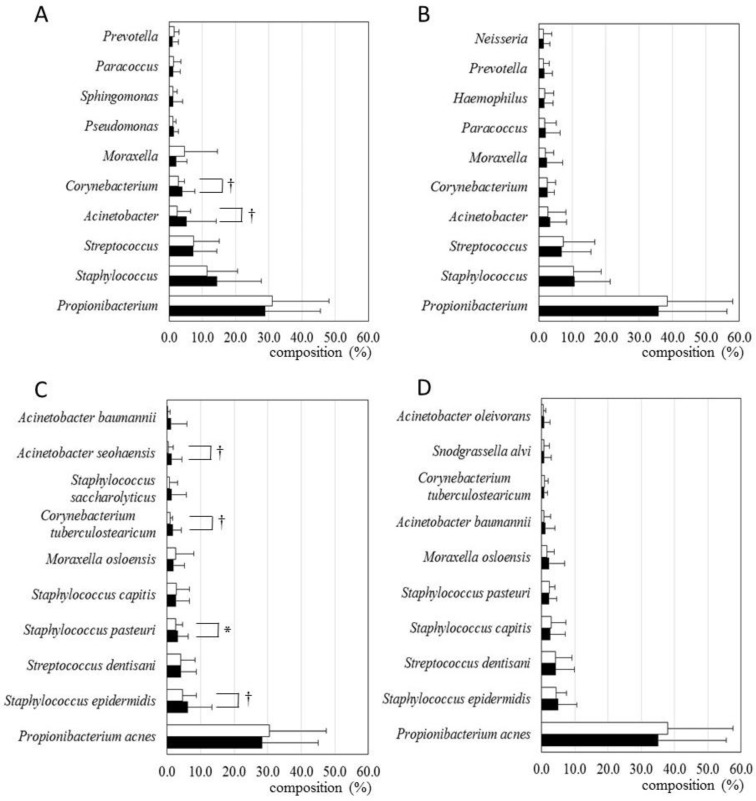
Relative composition of the 10 most abundant bacteria in the placebo and LC-plasma groups. Bars indicate the mean with standard deviations. Closed rectangles, 0 W; open rectangles, 8 W. (**A**) Genus composition in the placebo group. (**B**) Genus composition in the LC-plasma group. (**C**) Species composition in the placebo group. (**D**) Species composition in the LC-plasma group. † *p* < 0.10, * *p* < 0.05.

The relative quantity of the 10 most abundant genera and species were compared between the placebo group and the LC-plasma group (Figure 3). The relative quantity of major genera and species was similar between the two groups at the start of the study. After the intake period, the relative quantity of two major genera and four major species significantly or marginally changed in the placebo group. By contrast, the relative quantity of major genera and species did not change in the LC-plasma group.

Next, we also performed a more detailed analysis to investigate the changes in the relative quantity of each species and genus during the intake period. The results of species analysis showed that 4.11% of microbiota comprising 11 species significantly changed, and 10.92% comprising 13 species marginally changed in amount during the test period in the placebo group (Table S2). Only 0.35% comprising seven species and 0.27% comprising three species changed in amount during the test period in the LC-plasma group.

The results of the genus analysis suggested that 1.06% of microbiota comprising six genera significantly changed, and 10.24% comprising five genera marginally changed, in amount during the test period in the placebo group. Only 0.19% comprising five genera and 0.46% comprising four genera changed in amount during the test period in the LC-plasma group (Table S3).

**Figure 4:**
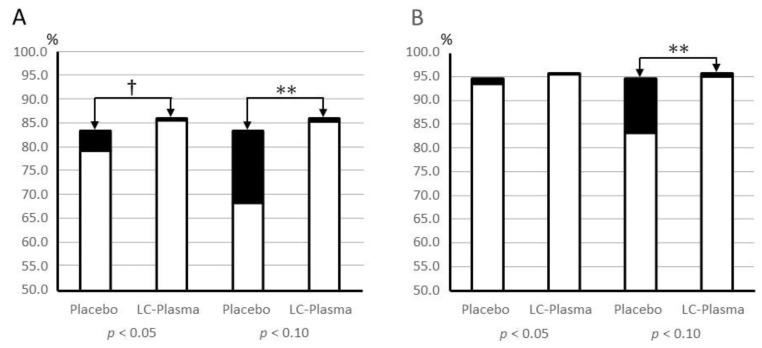
Comparison of the proportion of species (**A**) and genus (**B**) that changed (filled squares) or that did not change (open squares) during the test period. The sum of the relative composition of microbiota that significantly (*p* < 0.05) or marginally (*p* < 0.10) changed during the test period was compared between the placebo and the LC-plasma groups by Chi-square test. Data of each species and genus are shown in Tables S2 and S3, respectively. † *p* < 0.10, ** *p* < 0.01.

The chi-square test was used to compare the proportion of microbiota that significantly or marginally changed during the test period between the placebo group and the LC-plasma group (Figure 4). The results showed that the proportion of species and genera that marginally changed (*p* < 0.10) during the test period were significantly higher in the placebo group than that in the LC-plasma group. In addition, the proportion of species that significantly (*p* < 0.05) changed during the test period was marginally higher in the placebo group than in the LC-plasma group. No difference was observed in the proportion of genera that significantly changed during the test period. Taken together, these findings suggested that the microbiota composition was more robust in the LC-plasma group than in the placebo group.

### 3.4. LEfSe Analysis of Skin Micoroibota

**Figure 5:**
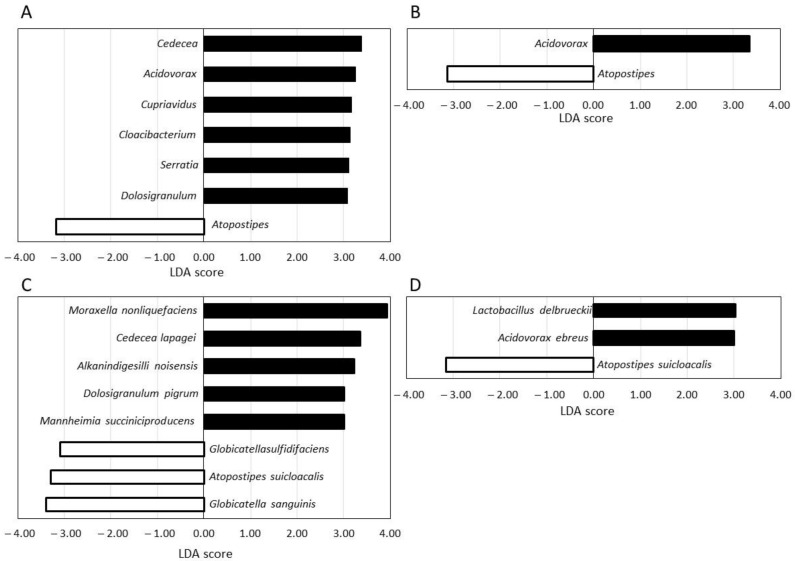
LEfSe results on the skin microbiome. Samples taken at 0 W and 8 W were compared in the placebo group and in the LC-plasma group. The significance level for LEfSe was set as *p* < 0.05, and only bacterial groups with an LDA score of >3 are displayed. (**A**) Comparison of genera in the placebo group. (**B**) Comparison of genera in the LC-plasma group. (**C**) Comparison of species in the placebo group. (**D**) Comparison of species in the LC-plasma group.

We also performed LEfSe analysis to identify changes in bacterial features during the test period between the 0 W and 8 W samples (Figure 5). In the placebo group, seven genera (Figure 5A) and eight species (Figure 5C) were identified as the candidates most likely to change during the test period. In the LC-plasma group, only two genera (Figure 5B) and three species (Figure 5D) were identified as candidates.

These results also suggested that microbiota composition was more robust in the LC-plasma group than in the placebo group.

### 3.5. Quantitative PCR Analysis of Skin Barrier-Related Genes

The relative expression levels of cytokine-encoding genes, TJ genes, and AMP genes are summarized in Table 3. To assess the effects of LC-plasma on skin immunity, we investigated the expression level of *IL1A*, *IL17A,* and *TGFB1*. No change was observed in *IL1A* expression in either group during the test period. By contrast, *TGFB1* was significantly down-regulated in the LC-plasma group, but no repression was observed in the placebo group during the test period. *IL17A* was not detected in either group.

To assess the effect of LC-plasma on TJ-related genes, we investigated the expression levels of claudin genes (*CLDN1*, *CLDN4*, *CLDN6*, *CLDN12*, and *CLDN18*), an occludin gene (*OCLN*), and TJ -associated protein genes (*ZO1*, *ZO2*, and *ZO3*) by quantitative PCR (qPCR). Among the five claudin genes, two (*CLDN1* and *CLDN12*) were significantly up-regulated in the LC-plasma group during the intake period. No significant increase was observed in the placebo group. Among the TJ-associated protein genes, *ZO1* was significantly up-regulated in the LC-plasma group, whereas a marginal increase in *ZO1* was observed in the placebo group. No difference was observed in the expression level of *ZO2* or *OCLN1* in either group. *CLDN6*, *CLDN18*, and *ZO3* were not detected.

To assess the effect of LC-plasma on AMP-encoding genes in general, we investigated six typical AMP genes. Overall, *BD3* was significantly up-regulated in the LC-plasma group but not in the placebo group during the intake period, and *BD1* was significantly up-regulated in both groups. No other AMP genes were up-regulated during the intake period.

Lastly, we performed post-hoc analysis of *TGFB1*, *CLDN1*, *CLDN12*, *ZO1*, *ZO2*, *BD1*, and *BD3*. None of these genes showed a significant difference in expression change between the placebo group and the LC-plasma group.

### 3.6. Skin Condition Assessments

The results of pigmentation, TEWL, and skin moisture contents are summarized in Table 4. The melanin index was significantly decreased in both groups over the intake period. The L * index was marginally increased in the LC-plasma group, but not in the placebo group. The a * and the Hb indexes significantly decreased in the LC-plasma group, but not in the placebo group. The b * index was marginally decreased in the placebo group. No change was observed in HbSO_2_, TEWL, or skin moisture content in either group during the test period. We also performed post-hoc analysis of the indexes of melanin, Hb, L *, a *, and b *. No index showed a significant difference between the placebo group and the LC-plasma group.

## 4. Discussion

In this study, we aimed to confirm the effects of LC-plasma in healthy humans. We primarily investigated the effects of LC-plasma on the skin microbiome that was not previously investigated in non-clinical experiments [11]. We studied facial skin microbiota, because the face is one of the most common loci where the composition of microbiota is related to disease or discomfort such as acne vulgaris.

The comparison of alpha diversity suggested that the intake of LC-plasma does not affect the Shannon–Weiner or Simpson indices. The comparison of the 10 most abundant genera and species suggested that 8 genera and 7 species were common between the placebo group and the LC-plasma group (Figure 2). These results were coincident with a previous report suggesting that the diversity of skin microbiome of an individual did not change for 2 years, which means the skin microbiome is qualitatively stable [21].

However, we found that the intake of LC-plasma affected the quantitative level of each microbiota during the test period. The relative quantities of two major genera and four major species were changed in the placebo group, while none of the relative quantities of major genera and species were significantly changed in the LC-plasma group. In addition, more detailed analysis of each genus and species showed that the total relative abundance of microorganisms that marginally changed quantitatively during the test period was significantly higher in the placebo group than that in the LC-plasma group. A similar tendency was observed in the significantly changed microorganisms. Lee et al. showed that the composition of the facial skin microbiome is affected by cosmetics, sebum, and hydration levels [22]. Our data suggested that the facial skin microbiome fluctuates quantitatively, probably due to environmental changes, and that the administration of LC-plasma may enhance the robustness of skin microbiota against daily external changes such as cosmetics. However, more analysis is necessary to confirm this, since the size of the subjects may not be large enough to detect the quantitative change of minor species.

The importance of robustness in the quantity of each microbiota has not yet been explored. Interestingly, we observed that the ratio of *S. aureus* to *S. epidermidis* was decreased only in the LC-plasma group (Table S2). Post-hoc analysis also suggested that this ratio was significantly higher in the placebo group than in the LC-plasma group (*p* = 0.021). *S. epidermidis* and *S*. *hominis* have been shown to secrete antimicrobial peptides that kill *S. aureus*, and transplantation of these species onto the skin of patients with atopic dermatitis leads to decreased colonization by *S. aureus* [23]. It has been also reported that butyric acid from *S. epidermidis* down-regulates the ultraviolet-induced pro-inflammatory IL-6 cytokine [24] and that the electricity produced by *S. epidermidis* caused significant growth attenuation and cell lysis of *P. acnes* [25]. Thus, robustness in the quantity of each microbiota might support microbe–microbe interactions between *Staphylococcus* species that are beneficial to the host. 

In the LEfSe analysis, more genera and species were identified as the candidates most likely to explain differences between 0 W and 8 W in the placebo group than in the LC-plasma group. Most of these candidates accounted for less than 0.1% of total microbiota and are not listed in Tables S2 and S3; however, several have been reported to be more abundant in the mucus membrane of head tissues, including *Dolosigranulum* in sinus [26], *Cloacibacterium* in laryngeal [27], and *Cedecea* in nasal membranes [28]. In addition, such microbiota have also been reported as important markers of disease or lifestyle, such as chronic rhinosinusitis, smoking, and adenoid hypertrophy. *Cupriavidus* has also been reported as a marker of cutaneous psoriasis [29]. It is possible that studies of the nasal, sinus, or psoriasis microbiome may reveal more significant effects of LC-plasma.

We also investigated the effect of LC-plasma on the expression of skin genes. The analysis of immunity genes suggested that *TGFB1* expression was down-regulated in the LC-plasma group, but not in the placebo group. TGF-βs are known to exert a wide range of biological effects on keratinocytes, such as growth inhibition and synthesis of plasminogen activator [30,31]. We also observed that the expression of two of four claudin genes and one of two ZO genes was up-regulated in the LC-plasma group but not in the placebo group. Claudins, a multigene family with at least 27 members in humans and mice, are the main components of TJs [32], which, as mentioned above, create the paracellular barrier [33,34]. *CLDN1* is the most abundant claudin in humans and is thought to be a key gene in human skin disease, especially atopic dermatitis [35,36]. The level of *CLDN1* is significantly lower in individuals with atopic dermatitis than in healthy adults. *ZO1* is a member of the membrane-associated guanylate kinase homolog (MAGUK) protein family and is thought to be involved in the proper organization of proteins within the TJ plaque [37]. *CLDN12*, which is also reported to be involved in TJ formation [38,39], showed the highest expression levels in this study. AMP genes were also up-regulated in this study, as well as in our previous study in mice [15]. Six AMP genes were previously up-regulated by LC-plasma in mice. In humans, *BD3* was up-regulated in the LC-plasma group (Table 3), and *BD2* was in the low-value layer of the LC-plasma group (data not shown). 

The results of pigmentation suggested that LC-plasma may affect the brightness and redness of the cheek. Redness (a *) was positively and strongly correlated with the Hb value (r^2^ = 0.68, *p* < 0.01, data not shown), as reported in other studies [40,41]. Fukuda et al. also suggested that skin redness is negatively correlated with blood flow rate [41]. It is possible that oral administration of LC-plasma affects the blood flow rate and increase the redness of the skin surface. Interestingly, a recent study suggested that the skin microbiome is influenced by skin pigmentation [42].

The mutual relationship between skin microbial dynamics and host gene expression remains poorly characterized. Our results suggested that the stability of the skin microbiome might be related to the expression of skin homeostasis-related genes. However, more analyses are necessary to clarify these issues because no difference in gene expression levels was observed between the placebo and LC-plasma groups. It is possible that studies on volunteers with diagnosed skin conditions might be useful to observe a greater difference in the skin microbiome and gene expression. In addition, more non-clinical studies are necessary to reveal the mechanism between the skin microbiome and host gene expression.

The main limitation of this study is that patients with skin diseases were not investigated. In the future, studies of patients with skin disorders caused by or associated with bacterial overgrowth, such as AD and acne vulgaris, will be performed. We are also planning studies on another anatomical part of the skin. In addition, studies regarding the effect of LC-plasma on the gut microbiome will be useful to clarify whether LC-plasma affects skin conditions via the skin–gut axis, an area that recently has been gaining attention [43,44]. In conclusion, our results suggest that oral administration of LC-plasma influences both the robustness of the skin microbiome and the expression of skin defense genes among healthy adults.

## Figures and Tables

**Table 1:** Exclusion criteria in this study.

(1)	Individuals who have diseases with medications.
(2)	Individuals who have continuously received medications within one month before the examination.
(3)	Individuals who have medical histories of serious disease of their liver, kidney, heart, lung, and blood.
(4)	Individuals who have comorbidity or medication history in their digestive organs.
(5)	Individuals whose systolic blood pressure is over 160 mmHg, or whose diastolic blood pressure is over 100 mmHg.
(6)	Individuals who have sever skin disorder.
(7)	Individuals who have severe anemia.
(8)	Individuals who might be allergic to test foods, or who might be seriously allergic to other foods, or medicaments.
(9)	Individuals who are pregnant, breastfeeding, or planning to be pregnant.
(10)	Individuals who are alcoholic or have mental disorder.
(11)	Individuals who will change their life style during the test period.
(12)	Individuals who have skin disease and need the application of medicine containing steroids or antibiotics to their face.
(13)	Individuals who have severe menopausal symptoms.
(14)	Individuals who cannot stop eating foods containing lactic acid bacteria.
(15)	Individuals who continuously took medicines for skin-condition-improvement within the last three month.
(16)	Individuals who use cosmetics that have strong effects on skin moisture or wrinkles.
(17)	Individuals who cannot keep from outdoor activities with the risk of getting sunburned during the test.
(18)	Individuals who had a surgery in their face within the past 6 months.
(19)	Individuals who are participating or participated in another clinical trial within the last 3 months.
(20)	Individuals who and whose family work for a company manufacturing or selling healthy foods, functional foods, and cosmetics.
(21)	Individuals who have smoking habitat.
(22)	Individuals who are judged unsuitable for this study by the investigator for other reasons.

**Table 2:** Baseline characteristics (mean ± S.D.) of the study subjects.

Item	Placebo	LC-Plasma	*p* Value *^1^
Number of subjects	35	33	
Gender	Male 10	Male 9	
	Female 25	Female 26	
Age	41.7 ± 8.6	40.2 ± 7.0	0.442
Weight (kg)	58.4 ± 10.9	56.2 ± 7.9	0.347
BMI *^2^ (kg/m^2^)	21.6 ± 2.9	21.1 ± 2.2	0.488
RBC *^3^ (×10^3^/mL)	4.72 ± 0.39	4.63 ± 0.37	0.331
WBC *^4^ (×10^3^/mL)	6.51 ± 1.82	6.13 ± 2.00	0.407
CD86 *^5^ (M.F.I.) *^6^	1378.7 ± 136.0	1402.3 ± 210.8	0.583
Skin moisture (A.U.) *^7^	50.7 ± 11.2	50.2 ± 14.0	0.871

* 1: *p* values calculated by Student’s t-test. * 2: body mass weight. * 3: red blood cell. * 4: white blood cell. * 5: cluster of differentiation, * 6: mean fluorescence intensity. * 7: arbitrary units.

**Table 3:** Relative expression of skin homeostasis-related genes.

Genes	Group	0 W	8 W	*p* Value (0 W vs 8 W)	*p* Value (Placebo vs LC-Plasma) ^*1^
Cytokine genes					
*IL1A*	Placebo	1.31 ± 0.91	1.59 ± 1.46	0.26	N.T.
	LC-Plasma	1.16 ± 0.64	1.59 ± 1.77	0.16	
*TGFB1*	Placebo	1.07 ± 0.44	1.01 ± 0.51	0.62	0.36
	LC-Plasma	0.95 ± 0.55	0.75 ± 0.27	0.03 *	
TJ genes					
*CLDN1*	Placebo	1.01 ± 0.32	1.02 ± 0.32	0.83	0.24
	LC-Plasma	0.91 ± 0.25	1.02 ± 0.28	0.05 *	
*CLDN4*	Placebo	1.05 ± 0.54	1.66 ± 3.00	0.25	N.T.
	LC-Plasma	1.02 ± 0.36	1.02 ± 0.34	0.99	
*CLDN12*	Placebo	3.10 ± 2.33	3.63 ± 1.85	0.23	0.67
	LC-Plasma	2.46 ± 1.95	3.24 ± 1.59	0.00 **	
*OCLN*	Placebo	1.24 ± 0.59	1.26 ± 0.52	0.87	N.T.
	LC-Plasma	1.18 ± 0.42	1.31 ± 0.62	0.27	
*ZO1*	Placebo	0.86 ± 0.39	1.04 ± 0.49	0.06 †	0.83
	LC-Plasma	0.72 ± 0.23	0.88 ± 0.44	0.03 *	
*ZO2*	Placebo	1.12 ± 0.55	1.17 ± 0.48	0.23	N.T.
	LC-Plasma	1.03 ± 0.33	1.11 ± 0.46	0.00 **	
AMP genes					
*BD1*	Placebo	1.20 ± 0.67	1.55 ± 0.80	0.01 **	0.34
	LC-Plasma	1.08 ± 0.56	1.30 ± 0.57	0.02 *	
*BD2*	Placebo	0.74 ± 2.26	0.79 ± 1.50	0.90	N.T.
	LC-Plasma	1.17 ± 1.89	1.57 ± 3.03	0.45	
*BD3*	Placebo	1.07 ± 0.66	1.19 ± 0.70	0.28	0.78
	LC-Plasma	0.98 ± 0.49	1.14 ± 0.54	0.05 *	

Data are shown as mean ± S.D. † *p* < 0.10, * *p* < 0.05, ** *p* < 0.01. *^1^: The change in each value during the test period was compared between placebo and LC-plasma groups as post-hoc analysis.

**Table 4:** Changes in skin indices. (Mean ± S.D.)

Indices	Group	0 W	8 W	*p* Value (0 W vs 8 W)	*p* Value (Placebo vs LC-Plasma) ^*1^
Melanin	Placebo	0.90 ± 0.20	0.88 ± 0.18	0.04 *	0.92
	LC-Plasma	0.96 ± 0.19	0.93 ± 0.19	0.02 *	
Hb	Placebo	1.01 ± 0.28	0.97 ± 0.22	0.20	0.32
	LC-Plasma	1.03 ± 0.23	0.95 ± 0.25	0.00 **	
HbSO_2_	Placebo	58.04 ± 5.52	56.92 ± 7.15	0.58	N.T.
	LC-Plasma	57.77 ± 7.21	58.40 ± 11.23	0.86	
L*	Placebo	64.79 ± 2.89	64.93 ± 2.51	0.51	0.47
	LC-Plasma	64.46 ± 3.46	64.81 ± 2.93	0.08 †	
a*	Placebo	7.00 ± 1.71	6.64 ± 1.29	0.06 †	0.46
	LC-Plasma	7.38 ± 1.46	6.83 ± 1.44	0.00 **	
b*	Placebo	16.52 ± 2.55	16.22 ± 2.44	0.08 †	0.57
	LC-Plasma	17.20 ± 2.28	17.03 ± 2.33	0.29	
TEWL (gm^−2^h^−1^)	Placebo	15.66 ± 5.60	15.99 ± 5.81	0.60	N.T.
	LC-Plasma	17.87 ± 5.95	18.71 ± 6.12	0.33	
Skin Moisture (A.U.) ^*2^	Placebo	47.95 ± 12.69	49.90 ± 12.25	0.20	N.T.
	LC-Plasma	48.22 ± 15.43	50.33 ± 15.48	0.25	

Data are shown as mean ± S.D. † *p* < 0.10, * *p* < 0.05, ** *p* < 0.01. * 1: The change in each value during the test period was compared between the Placebo and LC-plasma groups as a post-hoc analysis. * 2: Arbitrary unit.

## Data Availability

Data is contained within the article.

## Supplementary Materials

The following are available online at www.mdpi.com/article/10.3390/microorganisms9102029/s1, Table S1: Primer sequences used for quantitative PCR, Table S2: Relative composition of bacterial species that were significantly or marginally changed during the intake period, Table S3: Relative composition of bacterial genus that were significantly or marginally changed during the intake period.
